# Shaking *Takete* and Flowing *Maluma*. Non-Sense Words Are Associated with Motion Patterns

**DOI:** 10.1371/journal.pone.0150610

**Published:** 2016-03-03

**Authors:** Markus Koppensteiner, Pia Stephan, Johannes Paul Michael Jäschke

**Affiliations:** Department of Anthropology, University of Vienna, Vienna, Austria; University of Tokyo, JAPAN

## Abstract

People assign the artificial words *takete* and *kiki* to spiky, angular figures and the artificial words *maluma* and *bouba* to rounded figures. We examined whether such a cross-modal correspondence could also be found for human body motion. We transferred the body movements of speakers onto two-dimensional coordinates and created animated stick-figures based on this data. Then we invited people to judge these stimuli using the words *takete-maluma*, *bouba*-*kiki*, and several verbal descriptors that served as measures of angularity/smoothness. In addition to this we extracted the quantity of motion, the velocity of motion and the average angle between motion vectors from the coordinate data. Judgments of *takete* (and *kiki*) were related to verbal descriptors of angularity, a high quantity of motion, high velocity and sharper angles. Judgments of *maluma* (or *bouba*) were related to smooth movements, a low velocity, a lower quantity of motion and blunter angles. A forced-choice experiment during which we presented subsets with low and high rankers on our motion measures revealed that people preferably assigned stimuli displaying fast movements with sharp angles in motion vectors to *takete* and stimuli displaying slow movements with blunter angles in motion vectors to *maluma*. Results indicated that body movements share features with information inherent in words such as *takete* and *maluma* and that people perceive the body movements of speakers on the level of changes in motion direction (e.g., body moves to the left and then back to the right). Follow-up studies are needed to clarify whether impressions of angularity and smoothness have similar communicative values across different modalities and how this affects social judgments and person perception.

## Introduction

Onometopoeia, the relationship between the meaning of a word and its sound, is said to occur rarely in many languages [1]. However, despite representing different modalities, the sound of certain words (e.g., smooth sound) appears to share characteristics with certain shapes (e.g., smooth shape).

People show a high consensus when asked to associate certain made-up words with certain shapes. A famous example for this is the *takete*-*maluma* effect, which was named after an experiment devised by Köhler [2,3]. He invented the nonsense words “*takete*” and “*maluma*” and found that people preferably assigned *takete* to a spiky, angular figure and *maluma* to a rounded, curvy figure. Research using a different pair of words replicated these findings. People preferred the artificial word “*bouba*” as a name for a rounded shape and “*kiki*” as a name for a spiky shape [4]. More elaborated experiments using random combinations of certain vowels and consonants revealed that people tend to associate harsh sounding consonants such as t, k and p with spiky shapes and smooth sounding consonants such as m, l and n with rounded shapes [5].

The ability to link together cross-modal information seems very advanced at an early age. Young children, two or three years of age, have shown similar matching behaviors as adults when assigning made-up words with rounded vowels to rounded shapes and words with unrounded vowels to pointed shapes [6]. Other researchers demonstrated that children at that age are better able to learn verbs with a strong sound-symbol correspondence [7]. Also, this ability for cross-modal information linkage might be based on neurophysiology. While people with synesthesia experience an extreme form of associations, for instance when words give an impression of color [8] or induce a sensation of taste in the mouth [9], persons with an autism spectrum disorder are assumed to have great difficulty in making such associations. Correspondingly, autistic children have been shown to perform poorly at the *bouba*-*kiki* task [10]. Bearing all this in mind, it appears plausible that cross-modal matching may be biologically grounded and a useful ability of the human cognitive apparatus that, for instance, helps to integrate multisensory information [11,12].

Most researchers investigating the *takete*-*maluma* (or *bouba*-*kiki*) effect have used static cues with a rounded or an angular shape for their experiments. However, impressions of angularity or roundedness may also be imparted by dynamic cues. In common speech, for instance, movements are often described as smooth (as equivalent for rounded) or angular and such motion patterns can convey meanings of social relevance. For instance, ballet dancers portray “threatening” characters by adopting angular poses and displaying “angled movements”, while they portray “warm” characters by adopting rounded poses and displaying “rounded movements” [13]. Moreover, people are able to perceive motion in sounds. They differentiate between kinematic features of motion that have been transformed into the auditory domain [14] and recognize movements by the sounds they create [15,16]. Therefore, it is plausible to assume that people may be able to assign *takete* and *maluma* to cues that are created by human body movements.

It has already been shown that dynamic cues and motion kinematics convey a great deal of information ranging from people’s sex, their age, their emotional state, their dancing abilities, and their actual and perceived personality [17–23]. The current study uses such work as a starting point and intends to transfer the *takete-maluma* effect to dynamic cues embedded in the behavioral stream. We conducted a rating experiment in which we used short video clips of politicians giving a speech and transformed their body movements into animated stick-figures. Thus, in contrast to other *takete*-*maluma* studies our stimuli were not purely artificial but extracted from behaviors performed in a real life situation. We then asked participants to judge these stimuli on *takete*-*maluma*, *bouba*-*kiki* and several descriptors of body motion. In addition to collecting verbal assessments of body motion we also determined data driven descriptors of angularity and smoothness. Gesturing usually shows a certain similarity to the movements of a pendulum with movements in one direction often followed by a reverse movement or a change in direction at least (e.g., body moves to the left, then to the right, then back to the left etc.). Building on this our motion analyses divided the behavioral stream into single units of such movements, from which we extracted all local maxima (a maximum is reached before a movement changes direction), the velocity and the angles between successive changes of direction. Estimates of velocity were also used for a follow-up forced choice experiment where we presented stimuli reaching high and low values on this motion measure. Here, participants were asked to assign the stimuli to be either *takete* or *maluma*.

In accordance with previous research based on static cues we hypothesized that movements with a spiky, angular appearance are associated with *takete* (or *kiki*) and rounded or smooth movements are associated with *maluma* (or *bouba*). More precisely, we assumed that stick-figures displaying more changes in motion direction (i.e., movements and reverse movements), that produce movements with a higher velocity and sharper angles are more likely to be rated as angular and to be assigned to *takete*. By contrast, stick-figures displaying fewer changes in motion direction as well as producing a lower velocity and blunter angles should be rated as smoother and hence be assigned to *maluma*. In sum, we transferred the *takete*-*maluma* effect to body motion.

## Experiment I

We transformed the body movements of speakers into animated stick-figures and presented these stimuli to participants. The participants were asked to judge the stimuli on verbal descriptors of body motion and on the word-pairs *takete*-*maluma* and *bouba*-*kiki*. In addition to this, we created motion measures using the coordinate data obtained during behavior encoding and examined how these variables are related to the verbal descriptors of motion and ratings on the basis of the artificial word-pairs.

### Method

#### Ethics Statement

The Experiments were conducted in accordance with the Declaration of Helsinki (revised 1983) as well as local guidelines of the Faculty of Life Sciences, University of Vienna. In accordance with the Austrian Universities Act 2002 (UG2002), in place at the time the study was carried out, only medical universities were required to appoint ethics committees for clinical tests, application of medical methods, and applied medical research. As a result, no ethical approval was required for the present study. We did not collect personal data except age and gender. Experimental data, which were identified by numeric codes, were not personally traceable and therefore a written consent by the participants was not required.

#### Participants

Data collection was part of a practical course in human behavior research taking place at the University of Vienna’s Department of Anthropology. Students of this course asked 99 participants at locations throughout the university campus (60 females and 39 males; age *M* = 24 years, *SD* = 5.4) to take part in a rating experiment. People participated voluntarily in this experiment and were not reimbursed.

#### Stimulus Preparation

**Fig 1:**
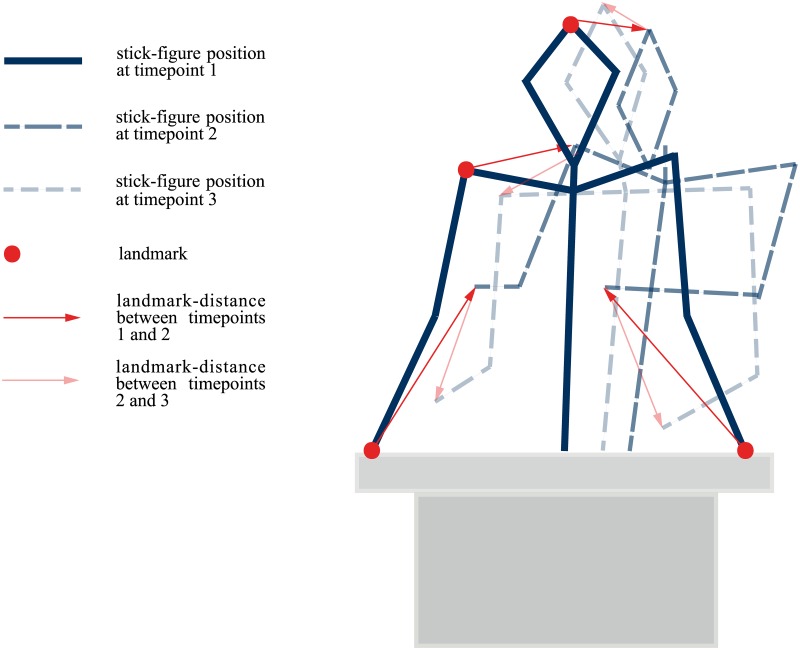
Landmark-positions of Stick-figure Stimuli at Different Points in Time.

Video clips of politicians giving a speech were used for motion capturing. From three parliamentary sessions in the German parliament we selected 60 of these video clips (30 male speakers and 30 female speakers) and extracted brief, randomly chosen video segments with an average length of 15 seconds (see Method in [24]). To record the body movements of the speakers we used the custom made software SpeechAnalyzer, which enables a user to access single pictures (i.e., frames) of a video clip. Starting with the first frame of each video clip landmarks were positioned on the speaker’s forehead, the hollow of the throat between the collar bones, ears, shoulders, elbows, hands, a spot in the middle of the body near the navel, and at the corners of the lectern (see Method in [20,21]). Afterwards videos were moved forward to the next frame and position shifts of the marked body regions were captured by replacing the landmarks. By repeating this procedure throughout the length of a video segment, body movements were turned into time-series of two dimensional landmark positions. Landmark displacement was supported by software routines based on optical flow. As this kind of automatic tracking was prone to error, in many cases landmarks had to be corrected by performing drag and drop operations with the computer mouse. Time series of these landmark coordinates were the basis for determining motion measures and creating stick-figure videos that served as abstract representations of the speakers’ body movements (Fig 1). To reduce workload we went through the movies in steps of three frames and filled in missing frames by linear interpolation. This guaranteed that stick-figure animations were on the same frame rate as the original videos.

#### Procedure

Potential participants were approached and asked if they had time to take part in a rating experiment. They were led to the laboratory, where they received instructions on how to use the rating program. Then they performed the rating tasks on their own (i.e., no experimenter present) using a computer-controlled interface. During the rating task participants watched a subset of 15 stick-figure movies each lasting 15 seconds. These stick-figure movies were presented in random order. Every video could be watched several times before starting the respective rating. Afterwards participants could proceed to the next video clip. On the left-hand side of the user interface stick-figure video clips were presented; on the right hand side rating scales on the basis of semantic differentials were displayed.

Participants were asked to judge the stimuli on the word pairs weich—hart (i.e., soft—hard), rund—eckig (i.e., rounded—angular), zackig—fließend (i.e., jerky—fluent) schnell—langsam (i.e., fast—slow), and the artificial words *takete*–*maluma* and *bouba*–*kiki*. Participants completed their ratings by dragging a trackbar control towards the item that they regarded as more appropriate. The scales were divided into 200 subunits, with -100 being the minimum value, +100 being the maximum value and 0 being the neutral position (i.e., similar to a visual analog scale). For the *takete*-*maluma* scale, this meant that the more a value was above 0 the more a stimulus “appeared *maluma*” and that the more a value was below 0 the more it “appeared *takete*”. The *bouba*-*kiki* scale was reversely arranged, which meant that the more a value was above 0 the more a stimulus “appeared *kiki*” and that the more a value was below 0 the more it “appeared *bouba*”.

#### Preparation of Coordinate Data

Coordinate data obtained during the motion capturing process was used to create data-driven motion descriptors. Previous research that also made use of the behavior encoding procedure applied here has shown that a small set of landmarks captures enough information to extract motion cues that can be used as predictors of people’s first impressions [20]. The motion descriptors of the present study were based on the same small set of landmark coordinates as in this previous work. We used information based on the forehead landmark to capture movements of the head, the landmark positioned on the left shoulder to capture all kinds of torso-movements (e.g., body moving sideways), and the landmarks of the left and right hand to capture all types of arm movements [20]. It was our intention to create overall descriptors of motion and not descriptors for each single landmark. For this reason, coordinates of the four selected landmarks were summed for each encoded frame and turned into single time series representing motion information (see S1 Dataset). Focusing on a small set of vital landmark-coordinates, which capture essential information, also reduces redundancy by not including landmarks, which capture similar information. To sum up, we obtained single columns of two-dimensional coordinates for each speaker, which contained information about the body movements they performed throughout the selected clips.

#### Extracting Maximum Distances

Coordinate data representing overall motion was the basis on which we created motion descriptors that served as measures of angularity and smoothness. In a first step, an algorithm was applied that turned the coordinates into distances between different points in time (i.e., frames). This algorithm uses the first frame as a reference point and calculates distances between these reference coordinates and successive coordinates (i.e., distance between frame one and two, distance between frame one and three etc.) until the calculated distance reaches a maximum. This maximum is stored and the coordinates of the maximum serve as a new reference point. Then the whole procedure starts again (i.e., distance between reference frame and reference frame plus one, distance between reference frame and reference frame plus two etc.). Thus, the algorithm yields time series of maximum distances that are determined on the basis of changing reference points (see Fig 1).

#### Number of Maxima

We assumed that the more often a stick-figure changes motion direction (e.g., body moves to the left and then back to the right) the more angular its body movements appear. As time-series of maximum distances (see above) give an estimate of such direction changes, we simply counted the number of such maxima.

#### Velocity

The faster a maximum distance is reached the faster a stick-figure changes motion direction and the more likely its movements should appear angular. Velocity was calculated by dividing a maximum distance by the time (i.e., number of frames) that was needed to reach this maximum. Summing up all the velocities (i.e., all maxima divided by the elapsed time) for one sequence and dividing it by the number of maxima gives the average velocity of body movements.

#### Average Angle between Motion Vectors

The algorithm described above not only can be used to determine maximum distances, but also to extract the direction vectors between successive reference points. Further, it is possible to determine angles between successive direction vectors of a sequence of behaviors. The mean of all these angles gives an estimate of the body movements’ angularity and smoothness. The larger this average angle is the more angular body movements should appear. The smaller this average angle is the smoother the body movements should appear.

#### Average Angle between Body Parts

Stick-figure animations are an abstract and parsimonious representation of body motion, but previous work investigating performances of ballet dancers has shown that static features (i.e., angular poses versus rounded poses) also have communicative value [13]. Hence, people’s impressions of angularity and roundness may also be influenced by appearance features. Sharp angles that originate from the positions of different body parts may interfere with perceptions of angularity created by body motion as well as affect classifications of the stick-figures as *takete* and *maluma*. Lifting the hands can lead to sharp angels between upper and lower arms and moving the hands sideways can lead to sharp angels between shoulders and upper arms. Hence, to create an estimate for the amount of static angularity we determined: (1) the angle between the right lower arm and the right upper arm, (2) the angle between the left lower arm and the left upper arm, (3) the angle between the right upper arm and the right shoulder, and (4) the angle between the left upper arm and the left shoulder. The four resulting angles were then summed for each encoded frame and this sum of angles was averaged across all frames of each stick-figure. Consequently, this yielded one single number for each stick-figure giving an estimate of the average angle between the mentioned body parts. The larger this measure is the more pointed angles (i.e., more angularity) are displayed during a sequence of behavior, the smaller this measure is the more blunt angles (i.e., less angularity) are displayed.

#### Statistical Analyses

Participants judged subsets of 15 stimuli drawn from the 60 stick-figure animations that were available. Animations were presented in random order while being divided into four subsets of stimuli. For each of these subsets we determined the reliability of the mean ratings by calculating intraclass correlations [25]. For further analyses we averaged the judgments of verbal motion descriptors as well as the judgments of *takete*-*maluma* and *bouba*-*kiki*. We expected verbal motion descriptors and data driven motion measures to be highly interdependent. Such intercorrelations undermine the interpretation of regression coefficients of multiple regressions. For this reason we used simple Pearson correlations to examine the relationships between the variables under investigation.

All statistical analyses were carried out in the program R [26].

### Results and Discussion

Participants of the rating-experiment watched and judged stick-figures that belonged to one of four subsets. Stimuli of the first subset were rated by 27 participants, stimuli of the second and the third subset were rated by 22 participants and stimuli of the last subset were rated by 28 participants. Average measure intraclass coefficients (ICC’s) for the ratings of all these groups are presented in Table 1. For most items the coefficients show moderate to strong agreement. ICC’s for *bouba*-*kiki* in the second and third group of stimuli were relatively low. For this reason results for *bouba-kiki* should be interpreted with some caution.

**Table 1:** Average Measure Intraclass Correlations for Verbal Descriptors of Body Motion and Ratings of *takete*-*maluma* and *bouba*-*kiki*.

	Subset 1	Subset 2	Subset 3	Subset 4
soft—hard	0.91	0.83	0.90	0.87
	[0.83,0.97]	[0.68,0.93]	[0.80,0.96]	[0.76,0.95]
rounded—angular	0.80	0.72	0.77	0.75
	[0.63,0.92]	[0.46,0.89]	[0.55,0.91]	[0.54,0.90]
jerky—fluent	0.77	0.85	0.68	0.80
	[0.58,0.91]	[0.72,0.94]	[0.39,0.87]	[0.62,0.92]
fast—slow	0.91	0.90	0.90	0.90
	[0.83,0.97]	[0.83,0.96]	[0.81,0.96]	[0.80,0.96]
*takete*–*maluma*	0.67	0.64	0.70	0.80
	[0.37,0.87]	[0.31,0.86]	[0.42,0.88]	[0.62,0.92]
*bouba*–*kiki*	0.65	0.29	0.48	0.62
	[0.34,0.86]	[-0.33,0.72]	[0.01,0.79]	[0.28,0.85]

*N* = 60; Numbers in brackets are 95% confidence intervals. People rated 15 randomly ordered stick-figures animations belonging to one of four subsets.

The correlation between average ratings of *takete*-*maluma* and ratings of *bouba*-*kiki* was high (*r*(58) = -0.72, *p* < 0.05). Thus, although bouba-kika yielded low ICC’s in part, on average both scales appear to convey similar information. Relationships of verbal motion descriptors with *takete*-*maluma* and *bouba*-*kiki* were also strong (Table 2). Consequently, the participants of our experiment tended to associate stick-figure movements they perceived as soft, rounded, fluent and slow with *maluma* or *bouba*, whereas movements that were perceived as hard, angular, jerky and fast were preferably associated with *takete* or *kiki*. In summary, these results indicated that body movements convey properties that also seem to be contained in the phonetic spelling of words or sounds in general. Moreover, the participants appeared not to differentiate between the verbal motion descriptors we used. Body movements that were perceived as angular were also perceived as hard, fast and jerky while body movements that were perceived as rounded were also perceived as soft, slow and fluent.

**Table 2:** Pearson Correlations of Verbal Descriptors of Body Motion with *takete*-*maluma* and *bouba*-*kiki*.

	soft—hard	rounded—angular	jerky—fluent	fast—slow
soft—hard	-			
			
rounded—angular	0.87***	-		
	[0.79,0.92]			
jerky—fluent	-0.84***	-0.88***	-	
	[-0.90,-0.74]	[-0.93,-0.81]		
fast—slow	-0.81***	-0.70***	0.76***	-
	[-0.88,-0.70]	[-0.81,-0.54]	[0.63,0.85]	
*takete*–*maluma*	-0.85***	-0.76***	0.80***	0.83***
	[-0.91,-0.76]	[-0.85,-0.63]	[0.68,0.88]	[0.72,0.89]
*bouba*–*kiki*	0.69***	0.72***	-0.73***	-0.63***
	[0.53,0.80]	[0.57,0.82]	[-0.83,-0.58]	[-0.77,-0.45]

*** *p* ≤ .001.

*N* = 60; Numbers in brackets are 95% confidence intervals.

Apart from having the stimuli assessed on verbal descriptors of motion we also used motion measures that were based on the coordinate data obtained during the process of behavior encoding. Correlations of the verbal descriptors with the motion measures we created revealed strong links with velocity and average angle (Table 3). Less pronounced but still remarkable relationships were found for the number of maxima. Our estimate of angularity derived from arm and shoulder positions (i.e., average angle between body parts) was only weakly related to the verbal descriptors of body motion. All in all, motion measures corresponded with people’s impressions of the stimuli’s body movements, while measures of overall angularity on the basis of body postures appeared to influence people’s impressions to a very small degree. For instance, measures of velocity went together with participants’ ratings of fast and slow. This clearly showed that the coordinate-based measure of velocity was in accordance with people’s ratings of velocity. It is also important to note that the motion measure average angle corresponded with people’s ratings on the scale rounded-angular. Thus, the sharper the angle of the stimuli’s motions the more likely their movements were rated as angular and less likely as rounded.

**Table 3:** Pearson Correlations of Verbal Descriptors of Body Motion with Measures based on Coordinate Data.

	soft—hard	rounded—angular	jerky—fluent	fast—slow
Number of Maxima	0.34**	0.38**	-0.40**	-0.30*
	[0.09,0.55]	[0.14,0.58]	[-0.60,-0.17]	[-0.52,-0.05]
Average Velocity	0.56***	0.51***	-0.42***	-0.61***
	[0.35,0.71]	[0.29,0.67]	[-0.61,-0.19]	[-0.75,-0.42]
Average Angle Motion Vector	0.53***	0.55***	-0.46***	-0.38**
	[0.32,0.69]	[0.34,0.70]	[-0.64,-0.23]	[-0.58,-0.14]
Average Angle between Body Parts	0.13	0.20	-0.16	-0.07
	[-0.13,0.37]	[-0.05,0.44]	[-0.40,0.09]	[-0.32,0.19]

* *p* < .05;

** *p* ≤ .01;

*** *p* ≤ .001.

*N* = 60; Number of Maxima is a measure of the amount of direction changes. Average Angle Motion Vector is the mean of all angles between direction vectors created by the stick-figure’s movements. Average Angle between Body Parts gives an overall estimate of the angularity between shoulders and upper arms as well as between upper arms and lower arms. Numbers in brackets are 95% confidence intervals.

Motion measures were strongly correlated with assessments of *takete*-*maluma* and *bouba*-*kiki* (Table 4). People tended to attribute stimuli with high values of velocity, a higher number of maxima and a sharper average angle to *takete* or *kiki*. This meant that stick-figures that showed fast movements, a great quantity of changes in direction (i.e., more maxima) and sharper angles (i.e., high average angle indicates sharper angles between direction vectors) between successive changes of direction were preferably rated as *takete* or *kiki*. Slow movements, a smaller quantity of changes in direction and blunter angles, on the other hand, were preferably associated with *maluma* or *bouba*. Estimates of angularity on the basis of shoulder and arm positions (i.e., average angle between body parts) only yielded a noteworthy relationship with judgements of *bouba-kiki*. Despite this, the results obtained clearly show that people’s impressions of *takete-maluma* and *bouba-kiki* were mainly guided by motion cues.

**Table 4:** Pearson Correlations of Motion Measures with Ratings of *takete*-*maluma* and *bouba*-*kiki*.

	Number of Maxima	Average Velocity	Average Angle Motion Vector	Average Angle Body Parts
*takete*–*maluma*	-0.41**	-0.51***	-0.49***	-0.12
	[-0.60,-0.17]	[-0.67,-0.29]	[-0.66,-0.26]	[-0.36,0.14]
*bouba*–*kiki*	0.34**	0.39**	0.38**	0.25*
	[0.10,0.55]	[0.15,0.58]	[0.14,0.58]	[0.00,0.48]

* *p* < .05;

** *p* ≤ .01;

*** *p* ≤ .001.

*N* = 60; Number of maxima measures the quantity of direction changes. Average Angle Motion Vector is the mean of all angles between direction vectors created by the stick-figure’s movements. Average Angle Body Parts gives an overall estimate of the angularity between shoulders and upper arms as well as between upper arms and lower arms. Numbers in brackets are 95% confidence intervals.

Previous research has shown that people assign *takete* to angular and *maluma* to rounded figures [4]. In contrast to such static cues motion patterns unfold over time. The movements and the gesturing of speakers are characterized by changes in direction. Simply put, movements run in one direction and then return. We broke down the behavioral stream into single units of such direction changes and applied motion measures to this data. We assumed that the faster (i.e., higher velocity) and the more often (i.e., higher number of maxima) changes in motion direction occur the more angular (i.e., sharper angles) and the less rounded movements should appear. The strong relationships of the verbal descriptors of body motion with the motion measures showed that velocity, the number of maxima and the average angle measure what they were intended to measure. Moreover, the verbal motion descriptors as well as the data driven motion measures proved to be related to *takete*-*maluma* and *bouba*-*kiki*. Thus, previous findings on the basis of these artificial words can also be transferred to perceptions of motion patterns.

## Experiment II

In experiment I the verbal descriptors of body motion (e.g., rounded versus angular) were presented in parallel with the rating scales for *takete*-*maluma* and *bouba*-*kiki*. Therefore, it is conceivable that the ratings influenced each other, and this could in part explain the results obtained. For this reason we performed a second experiment, which served as a retest of the first one and was based on a subselection of the available videos. Selection criterion was the velocity of the stick-figures’ movements because velocity yielded the most convincing results in experiment I. In a forced choice experiment combinations of stick figure animations reaching high and low values on velocity were presented simultaneously on a computer screen and participants were asked to choose those animations they perceived as being more *takete* or more *maluma*.

### Method

#### Participants

Again students of a practical course in human behavior research recruited participants for rating experiments. The participants included 46 students (24 females and 22 males; age *M* = 25.1 years, *SD* = 3.6) of the University of Vienna. Participants were not reimbursed for taking part in the experiment.

#### Stimulus Preparation

As stimuli for a forced choice experiment we selected two subsets of the stick-figure animations that we had presented in the first experiment. We first ranked all stick-figures according to their values on the motion measure velocity and then chose ten stick-figure animations with low values and ten stick-figure animations with high values on this motion measure. In other words, we chose stick-figure animations as stimuli that showed opposing patterns of motion (e.g., fast movements with sharp angles versus slow movements with blunt angles).

#### Procedure

The procedure of participant recruitment was the same as in the first experiment. Students of the University of Vienna were approached personally and asked if they were willing to take part in a brief rating experiment. In Experiment II the computer-controlled interface either showed the word *maluma* or the word *takete* above two stick-figure animations that were presented simultaneously. One of the stick-figure animations was randomly chosen out of the high-rankers on the motion measure velocity, while the other stick figure was randomly chosen from the low rankers. Participants had to click on the stick-figure animation that appeared to better fit with the word that the program presented (i.e., *takete* or *maluma*). The experimental session for one participant comprised ten rounds. For each round *takete* or *maluma* was chosen randomly by the program as well as the side on which the “low and high ranked” stick-figure animations were presented. We only used the word pair *takete*-*maluma* for this experiment, because we obtained better results for it in the first experiment than for the word pair *bouba*-*kiki* (see Tables 1 and 4).

#### Statistical Analyses

During the rating sessions pairs of stick-figure animations were presented. If people randomly chose one of the two alternatives, we would expect them to have half of the answers correct (i.e., *p* = 0.5). To test whether people scored significantly higher than this we calculated binomial tests for all trials as well as additional binomial tests for the *takete* and the *maluma* condition.

### Results and Discussion

We expected people to preferably assign stick-figures with high values on the motion measure velocity (also meaning more maxima and sharper angles) to *takete*, and stick-figures with low values on velocity to *maluma*. “Correct” answers (i.e., answers that met our expectations) per rater ranged from one to ten with the most frequent number being ten (i.e., all answers correct). Thirty-three of 46 raters gave more than five correct answers.

Overall, there were 460 trials and people “correctly” assigned *takete* or *maluma* to the different stick-figures in 326 cases. Consequently, in 71% (95% CI [66%, 75%]) of all cases people made a choice that was in line with our predictions. This is highly significant according to an exact binomial test (*p* < 0.001). In 246 trials people were asked to assign *takete* to one of the two presented stick-figures. In 178 cases they did this according to our expectation and thus assigned 72% (95% CI [66%,78%]) of those stick-figures to *takete* that displayed angular, jerky, and fast movements (yields *p* < 0.001 in an exact binomial test). In 214 trials people chose a stick-figure on the basis of the word *maluma*. In 148 (i.e., 69%; 95% CI [63%, 75%]) of these trials they chose those stick-figures that showed less angular, slow, and smooth movements (yields *p* <0.001 in an exact binomial test).

Results of this experiment supported those obtained in the first one. People tended to assign the artificial word *takete* to patterns of motion characterized by high velocity. The word *maluma*, on the other hand, was assigned to patterns of low velocity. This further underlined that our motion measures capture qualities of the behavioral stream that correspond to patterns that are also inherent in the words *takete* and *maluma*.

## General Discussion

We captured the body movements of speakers and turned their performances into short video clips of animated stick-figures. These videos were then shown to participants who judged them on several descriptors of body motion and the artificial words *takete*-*maluma* and *bouba*-*kiki*. In addition to this we extracted different features of motion (e.g., velocity of the body movements) from the coordinate data we recorded during the motion capture process.

We found that people’s assessments of the stick-figures’ motion patterns (e.g., angular or fast) were related to data-driven measures of motion (e.g., velocity). This supports that the measures we devised are useful estimators of certain motion qualities and that it is possible to translate some aspects of how people perceive movements into measureable units. In other words, common language descriptions of body movements as smooth, jerky or expansive are reflected in distance measures between landmarks positioned on a human body and the dynamic changes of these distances. Single units of motion were the distances between two successive changes in motion direction (e.g., a body lifting, followed by lowering of the body, followed by a lifting again equals three movements). The relatively high agreement between the landmark-based measures of motion and the verbal descriptors (e.g., rounded–angular) hints that such movement units also guide people’s perceptions of motion. They may perceive body movements as sequences of changes in direction.

People are able to extract common patterns from different sources of information. For instance, they predominately assign artificial words containing harsh sounding consonants such as *takete* to spiky figures and words containing smooth sounding consonants such as *maluma* to rounded figures [5]. In this study we revealed that such artificial words also seem to be associated with cues perceptible in human body movements. For instance, the participants in our experiments preferably linked faster movements to *takete* and slower movements to *maluma*. Thus, the position shifts of a human body appeared to create patterns that correspond with patterns inherent in the words *takete* and *maluma* (or *bouba* and *kiki*). Although movements can be described as angular or rounded—qualities akin to shapes—such shape-like features in motion only become visible over time. Previous research has already found that the *takete*-*maluma* effect is not only confined to sound-shape associations [27]. Findings of this study indicate that it may be extended to patterns that arise from the behavioral stream of a speaker’s moving body.

Some researchers have considered that cross-modal correspondences are learned through implicit processes that integrate information from different sources in one’s environment [5, 12]; others have claimed that those associations have their roots in human evolution and therefore constitute a universal human ability [28]. In this study we provided no evidence that speaks clearly in favor for one of these positions. However, it appears conceivable that cross-modal associations have a biological foundation that is cultivated through interactions with environmental factors during ontogeny. For this reason, some cross modal associations may be made with greater ease because they occur more often in combination. Movements are visual cues but they may also produce a sound and when people communicate, gesturing and speech are coupled [29,30]. In natural and social environments cues from different modalities appear together and for this reason people are experienced in linking them together. Indeed, when body movements, such as drawing movements or walking actions, are translated into the auditory domain a great deal of the original information is preserved and still recognized by people [15,16]. With the approach introduced in this study it may be possible to show that such a coupling is also based on similar cross-modal patterns of angularity and smoothness and that speakers who display movements that are associated with *takete* also produce *takete*-like vocal cues.

In primates, vocalizations in aggressive encounters or alarm vocalizations are not arbitrarily structured but usually are loud and characterized by sharp and abrupt onsets with variations in frequency and amplitude. More gradual onsets and more harmonic frequency spectra are produced when primates seek close contact with companions. Similar patterns were found for humans [5,31]. Consequently, on an abstract level features described as sharp or abrupt may be communicating aggression, whereas features described as smooth and rounded may be communicating friendliness. For visual cues this has already been shown. People prefer objects with a more curved contour to objects with sharp transitions in contour because the latter appear to be communicators of threat [32]. Moreover, poses in ballet intended to display “warm” characters comprise more “round” elements, whereas poses intended to display “threatening” characters contain more angularity [13]. Therefore, a follow-up of the work presented here should investigate whether jerky body movements classified as *takete* are also classified as aggressive and whether rounded movements classified as *maluma* also appear friendly. Previous findings on body motion already revealed that motion cues are related to perceptions of friendliness [20,23]. Linking this to our findings on the *takete-maluma* effect could reveal that common patterns perceived in different modalities may have a similar impact on impression formation. For instance, *takete*-like movements and corresponding patterns of *takete*-like sounds may lead to similar attributions of friendliness.

## Conclusions

People recognize similarities in information from different modalities. One famous example for this is the *takete*-*maluma* (or *bouba*-*kiki*) effect, which has been replicated in many cultures. We transferred this effect to dynamic cues and found that people are able to link patterns created by moving bodies of speakers to patterns inherent in the artificial words *takete* (or *kiki*) and *maluma* (or *bouba*). *Takete* is associated with angularity and *maluma* with smoothness—features that also seem to become visible in the behavioral stream. This indicates that human nonverbal communication is impacted by simple patterns that can be matched across different sources of information. Given that the *takete*-*maluma* effect is stable throughout many cultures it appears conceivable that the correspondences we found constitute a level of communication that may be a part of many cultures. Future work should investigate if the results obtained here can be replicated in different cultures, increase the level of abstraction to have better control over information conveyed by shape and body postures (e.g., by mapping motion patterns found here onto one single moving dot), and build a bridge to research on the social impact of motion cues and person perception in general.

## Supporting Information

S1 Dataset R code and corresponding data to perform all statistical analyses presented in the manuscript (requires R; download R software from http://cran.r-project.org). (ZIP)
